# Supplementary material for: DNA binding and RAD51 engagement by the BRCA2 C-terminus orchestrate DNA repair and replication fork preservation
Source: Nat Commun. 2023 Jan 26;14:432. doi: 10.1038/s41467-023-36211-x (PMC9879961; doi:10.1038/s41467-023-36211-x)
Supplement: Supplementary file 1 — Supplementary Information [file 41467_2023_36211_MOESM1_ESM.pdf]

DNA Binding and RAD51 Engagement by the BRCA2 C-terminus Orchestrate DNA Repair and Replication Fork Preservation

Youngho Kwon[1,*], Heike Rösner[2,*], Weixing Zhao[1], Platon Selemenakis[3, 4], Zhuoling He[1], Ajinkya S. Kawale[1,5], Jeffrey N. Katz[1], Cody M. Rogers[1], Francisco E. Neal[1], Aida Badamchi Shabestari[1], Valdemaras Petrosius[2], Akhilesh K. Singh[6,7], Marina Z. Joel[6,8], Lucy Lu[6], Stephen P. Holloway[1], Sandeep Burma[1,9], Bipasha Mukherjee[9], Robert Hromas[10], Alexander Mazin[1], Claudia Wiese[3,**], Claus S. Sørensen[2,**], and Patrick Sung[1,**]

[1] Department of Biochemistry and Structural Biology and Greehey Children's Cancer Research Institute, University of Texas Health Science Center at San Antonio, Texas 78229, USA.

[2] Biotech Research and Innovation Centre, Faculty of Health and Medical Sciences, University of Copenhagen, DK-2200 Copenhagen N, Denmark.

[3] Department of Environmental and Radiological Health Sciences, Colorado State University, Fort Collins, Colorado, USA.

[4] Present address: Department of Cancer Biology, University of Texas MD Anderson Cancer Center, Houston, TX, USA.

[5] Present address: Massachusetts General Hospital Cancer Center, Harvard Medical School, Charlestown, MA 02129, USA.

[6] Department of Molecular Biophysics and Biochemistry, Yale University School of Medicine, New Haven, CT, USA.

[7] Present address: GentiBio Inc. 150 Cambridgepark Dr, Cambridge, MA, 02140, USA.

[8] Present address: Johns Hopkins University School of Medicine, Baltimore, MD, 21205, USA.

[9] Department of Neurosurgery, University of Texas Health Science Center at San Antonio, San Antonio, TX 78229, USA.

[10] Department of Medicine, University of Texas Health at San Antonio, 7703 Floyd Curl Drive, San Antonio, TX, 78229, USA.

[*] These authors contributed equally.

[**]Correspondence: claudia.wiese@colostate.edu, claus.storgaard@bric.ku.dk, sungp@uthscsa.edu

----

Includes:

Supplementary Figures 1 through 5
Supplementary Tables 1 and 2
Supplementary References

Supplementary Fig. 1

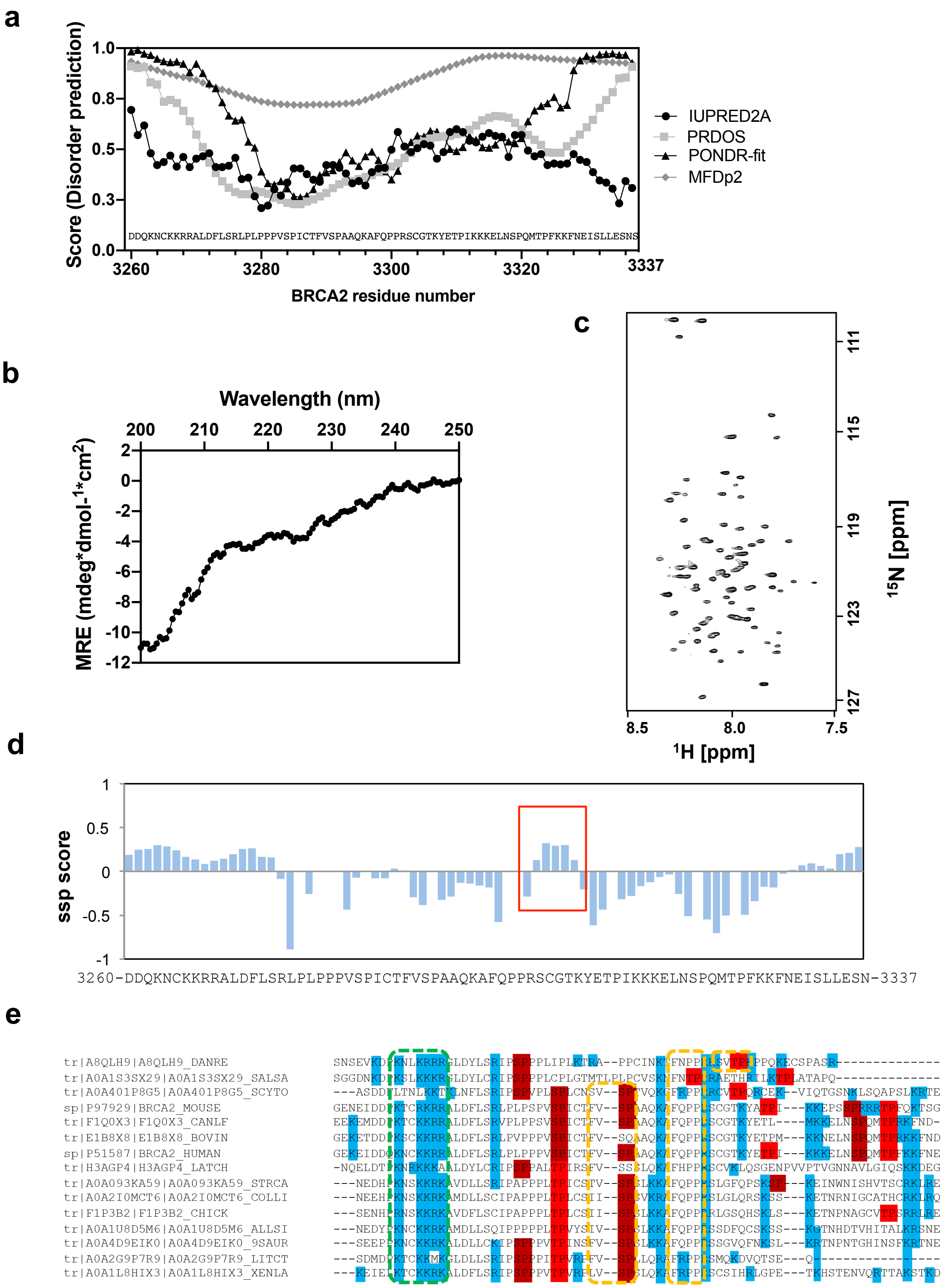

Supplementary Fig. 1. Disordered conformation of BRCA2 CTRB and identified PTM motifs from GPS web server.

a, Predicted disorder for the BRCA2-CTRB fragment (3260-3337). Four different disorder prediction algorithms (IUPRED2A, PONDR-fit, PRDOS, MFDp2) were used to calculate the degree of disorder. Source data are provided as a Source Data file.

b, Far UV-CD spectrum of the BRCA2- CTRB-derived polypeptide showing that it consists of random coil predominantly. Circular dichroism was recorded using 10 µM BRCA2 CTRB (3260-3337) in 1 x PBS, pH 6.0, 10 mM DTT, at 25°C. Source data are provided as a Source Data file.

c, ^{1}H-^{15}N-HSQC spectrum of BRCA2-CTRB: The narrow peak dispersion in the proton dimension is clearly an indication for a predominantly disordered conformation in the BRCA2-CTRB. The ^{1}H-^{15}N-HSQC NMR spectrum was recorded using 20 µM ^{1}H-^{15}N-labelled BRCA2 CTRB (3260-3337) in 1 x PBS, pH 6.0, 10 mM DTT, at 5°C.

d, Ssp-scores[1] for the BRCA2 CTRB polypeptide. Positive values indicate a preference for helical conformations, while negative values should be interpreted as an extended conformation. The majority of residues adopts a negative value unravelling the extended nature of the fragment. Two short regions of transient helicity can be found from residue 3260-3269 and residue 3300-3305 (as also highlighted by the red box). Source data are provided as a Source Data file.

e, Possible functional and post-translational modification (PTM) motifs in the CTRB. Representative excerpt of the alignment of vertebrate CTRB core fragment. Potential CDK phosphorylation sites are colored in red. All positively charged residues are colored in blue. The tandem FXXP motifs are highlighted within the yellow box. The cluster of highly conserved positive charges is highlighted within the green box.

Supplementary Fig. 2

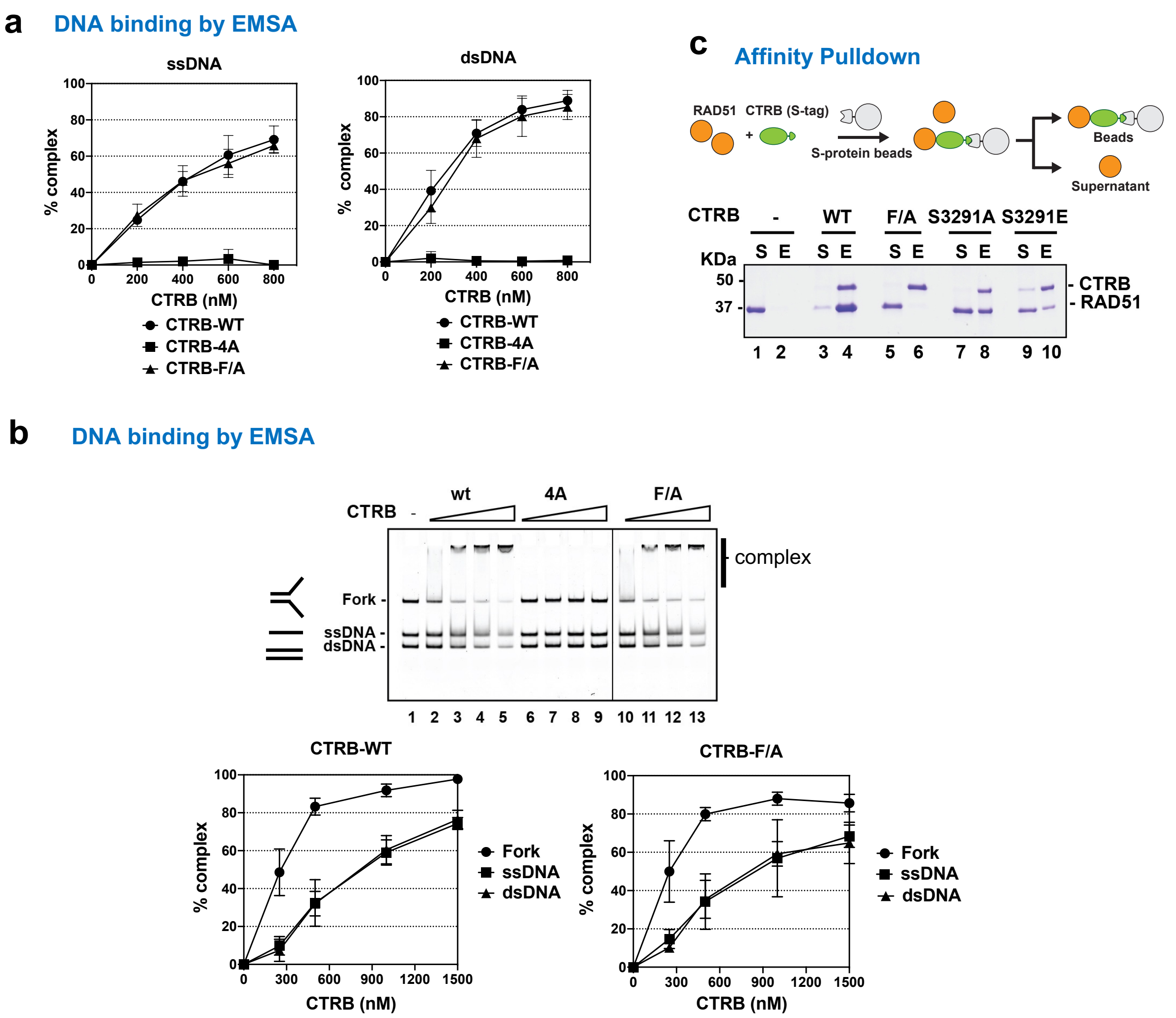

d

Sequence (3265–3330)		RAD51 interaction
CKKRRALDFLSRLPLPPPVSPICTFVSPAAQKAFQPPRSCGTKYETPIKKKELNSPQMTPFKKFNE	(3265-3330)	+
CKKRRALDFLSRLPLPPPVSPICTFV-----KAFQPPRSCGTKYETPIKKKELNSPQMTPFKKFNE	Δ(3291~3295)	+
CKKRRALDFLSRLPLPPPVSPICTFVSPAAQ-----PRSCGTKYETPIKKKELNSPQMTPFKKFNE	Δ(3296~3300)	−
CKKRRALDFLSRLPLPPPVSPICTFVSPAAQKAFQP-----TKYETPIKKKELNSPQMTPFKKFNE	Δ(3301~3305)	−
CKKRRALDFLSRLPLPPPVSPICTFVSPAAQKAFQPPRSCG-----PIKKKELNSPQMTPFKKFNE	Δ(3306~3310)	+
CKKRRALDFLSRLPLPPPVSPICTFVAPAAQKAFQPPRSCGTKYETPIKKKELNSPQMTPFKKFNE	S3291A	+
CKKRRALDFLSRLPLPPPVSPICTFVSPAAQAAAQPPASCGTKYETPIKKKELNSPQMTPFKKFNE	K3296A/F3298A /R3302A	−
CKKRRALDFLSRLPLPPPVSPICTFVSPAAQAAFQPPASCGTKYETPIKKKELNSPQMTPFKKFNE	K3296A/R3302A	+
CKKRRALDFLSRLPLPPPVSPICTFVSPAAQAAFQPPRSCGTKYETPIKKKELNSPQMTPFKKFNE	K3296A	+
CKKRRALDFLSRLPLPPPVSPICTFVSPAAQKAFQPPASCGTKYETPIKKKELNSPQMTPFKKFNE	R3302A	+
CKKRRALDFLSRLPLPPPVSPICTFVSPAAQKAAQPPRSCGTKYETPIKKKELNSPQMTPFKKFNE	F3298A	−

Supplementary Fig. 2. Biochemical activities of the CTRB.

a, Quantification of DNA binding data from the experiment shown in Fig. 2c. (n=4 biologically independent experiments for WT; n=3 for F/A and 4A; mean value ± SD). Source data are provided as a Source Data file.

b, The indicated CTRB species were incubated with ssDNA, dsDNA, and DNA fork and subject to EMSA. (n=6 biologically independent experiments for CTRB WT; n=3 for F/A and 4A; mean value ± SD). The samples were derived from the same experiment and processed in parallel. Source data are provided as a Source Data file.

c, Pulldown assay to examine the effect of the CTRB-F/A, CTRB-S3291A, or CTRB-S3291E mutation on RAD51 interaction. The supernatant (S) containing unbound proteins and SDS eluate of the anti-S tag resin (E) were analyzed by SDS-PAGE and Coomassie blue staining. The samples were derived from the same experiment and processed in parallel. The experiment was repeated three times with similar results. Source data are provided as a Source Data file.

d, Mapping of the RAD51 interaction domain in the CTRB. GST-TR2 (BRCA2 residues 3265-3330)[2] and various point and truncation mutant derivatives were tested for RAD51 interaction using glutathione resin.

Supplementary Fig. 3

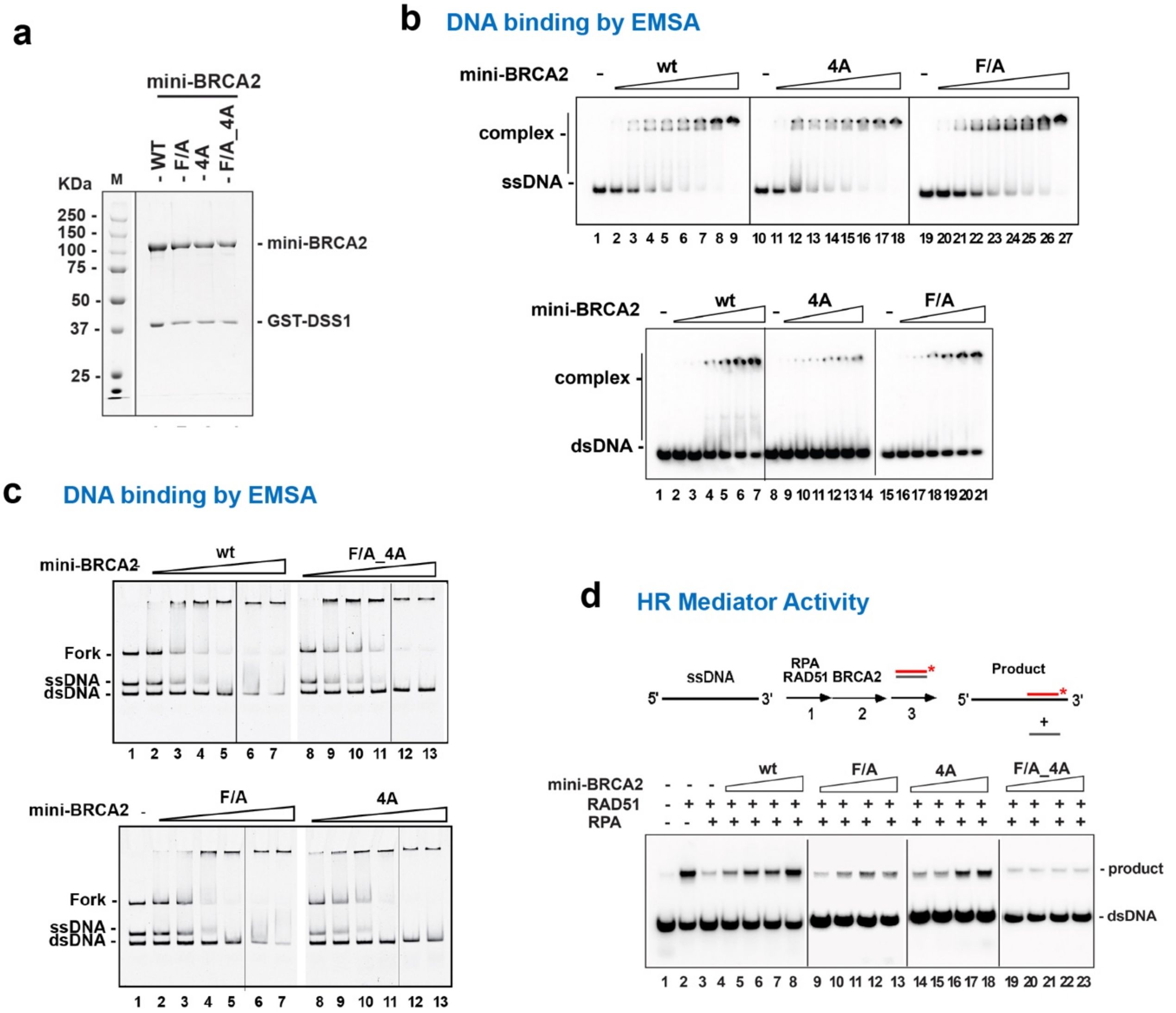

Supplementary Fig. 3. Biochemical activities of mini-BRCA2/DSS1 harboring F/A, 4A, or F/A-4A mutation.

a, SDS-PAGE of purified mini-BRCA2 protein species. The experiment was repeated three times with similar results. Source data are provided as a Source Data file.

b, EMSA performed with the mini-BRCA2/DSS1 proteins with $^{32-}$P-labeled ssDNA or dsDNA (13, 25, 38, 50, 63, 75, 100, 200 nM of mini-BRCA2/DSS1 for ssDNA; 25, 50, 100, 150, 200, 300 nM of mini-BRCA2/DSS1 for dsDNA). The samples were derived from the same experiment and processed in parallel. (n=3 biologically independent experiments). Source data are provided as a Source Data file.

c, EMSA performed with mini-BRCA2 proteins (30, 60, 90, 180, 270, 360 nM) with a mixture of Cy-5-labeled ssDNA, dsDNA, and DNA fork. The samples were derived from the same experiment and processed in parallel. The experiment was repeated twice with similar results. Source data are provided as a Source Data file.

d, Testing of mini-BRCA2/DSS1 (50, 100, 200, 300 nM) species for HR mediator activity with RAD51 (2 μM) and RPA (500 nM), as indicated. The samples were derived from the same experiment and processed in parallel. (n=4 biologically independent experiments). Source data are provided as a Source Data file.

Supplementary Fig. 4

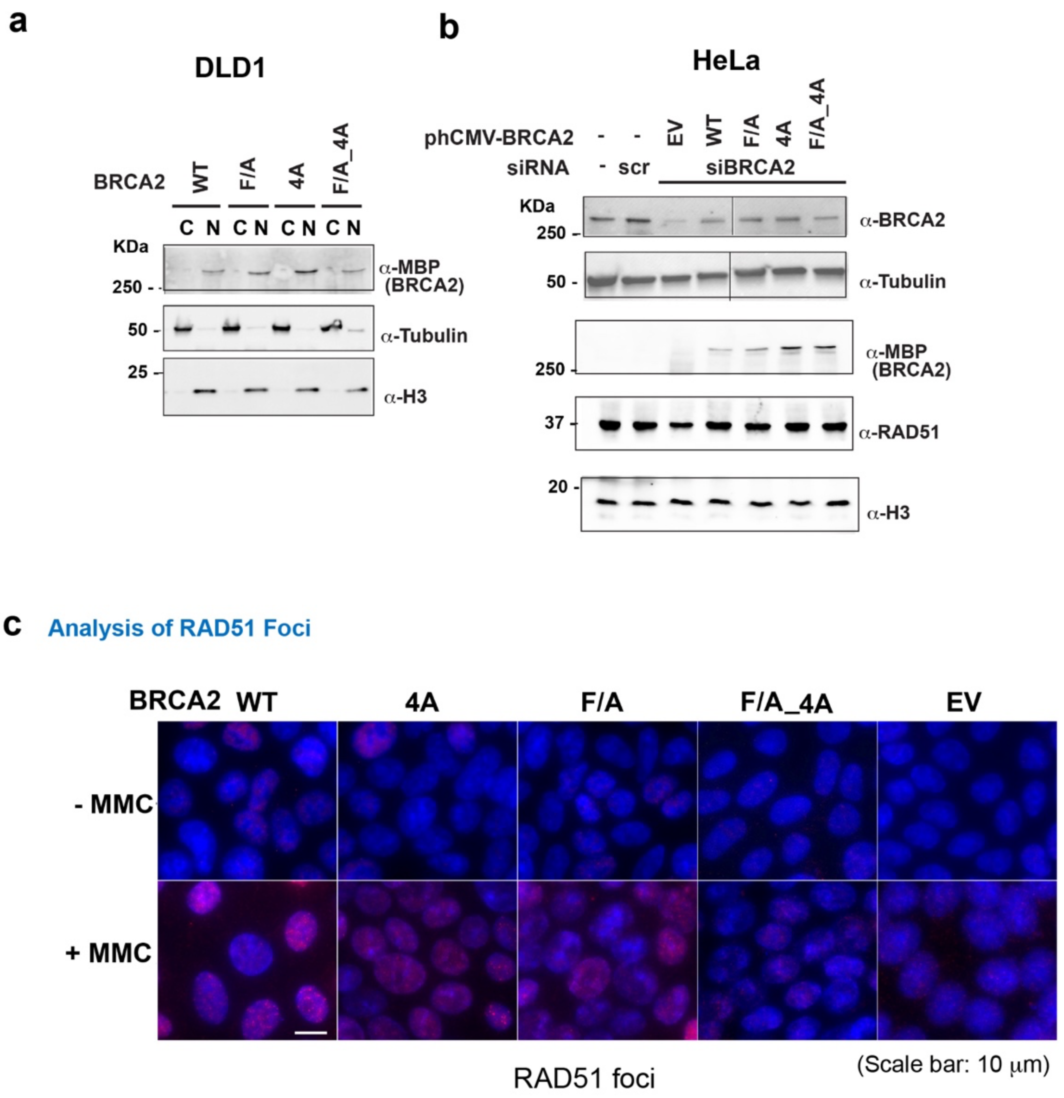

Supplementary Fig. 4. Expression, cellular localization, and RAD51 foci analyses of BRCA2 and mutants.

a, Western blot analysis of cytoplasmic (C) and nuclear (N) fractions prepared from DLD-1 cells expressing either wild type BRCA2 or its CTRB mutant variants. The samples were derived from the same experiment and processed in parallel. These samples were analyzed in two gels, with the top panel (α–MBP (BRCA2)) from one gel and the bottom two panels (α–Tubulin, α–H3) from another gel. The experiment was repeated three times with similar results. Source data are provided as a Source Data file.

b, Western blot analysis to verify ectopic expression of BRCA2 and CTRB mutants in HeLa cells. MBP-tagged BRCA2 species were expressed after treatment of HeLa DR-GFP cells with BRCA2 siRNA (siBRCA2) or scrambled RNA (scr). The samples were derived from the same experiment

and processed in parallel. These samples were analyzed in two gels, with the upper two panels (α–BRCA2 and α–Tubulin (loading control)) from one gel and the bottom three panels (α–MBP, α–RAD51, and α–H3 (loading control)) from another gel. The experiment was repeated twice with similar results. Source data are provided as a Source Data file.

c, Representative micrographs of RAD51 foci (red) in the nuclei (blue: DAPI staining) of DLD1-cells expressing BRCA2 or the indicated CTRB mutant after 1 μM MMC treatment for 16 h. Bar: 10 μm. EV: empty vector (phCMV-2MBP), NT: not-treated. The samples were derived from the same experiment and processed in parallel.

Supplementary Fig. 5

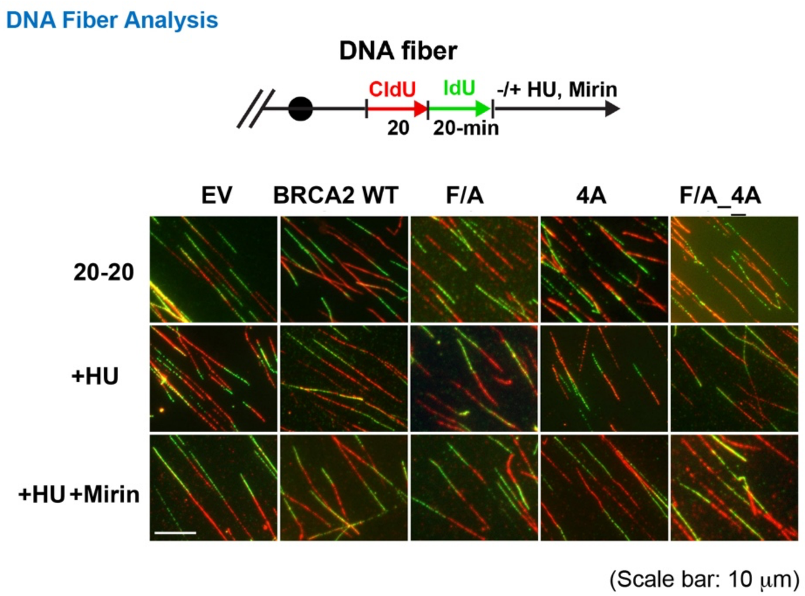

Supplementary Fig. 5. Analysis of impact of BRCA2 CTRB mutations on replication fork stability

Representative micrographs of CldU/IdU replication tracts in DLD1 cells expressing the indicated BRCA2. Cells were pulse-labeled 20 min each by CldU and IdU and then exposed to HU with or without mirin. The samples were derived from the same experiment and processed in parallel.

Supplementary Table 1: Sites of potential post-translational modification as predicted by the GPS web server[3,4].

ID	Position	Code	Enzyme	Peptide	Score	Cutoff
CTRB	36	K	Cullin RING/SCF/F-box	ICTFVSPAAQKAFQPPRSCGT	0.907	0.6726
CTRB	67	K	Cullin RING/SCF/F-box	LNSPQMTPFKKFNEISLLESN	0.7666	0.6726
CTRB	36	K	APC_C	ICTFVSPAAQKAFQPPRSCGT	0.9992	0.0161
CTRB	47	K	APC_C	AFQPPRSCGTKYETPIKKKEL	0.9963	0.0161
CTRB	53	K	APC_C	SCGTKYETPIKKKELNSPQMT	0.9962	0.0161
CTRB	54	K	APC_C	CGTKYETPIKKKELNSPQMTP	0.9968	0.0161
CTRB	55	K	APC_C	GTKYETPIKKKELNSPQMTPF	0.9995	0.0161
CTRB	66	K	APC_C	ELNSPQMTPFKKFNEISLLES	0.9988	0.0161
CTRB	67	K	APC_C	LNSPQMTPFKKFNEISLLESN	0.989	0.0161
CTRB	67	K	Sumoylation Nonconcensus	PQMTPFKKFNEISLL	3.62	3.32
CTRB	71 - 75		SUMO Interaktion	PFKKFNEISLLESN*****	40.379	29.92
CTRB	64	S	AGC/PKA	IKKKELNSP QMTPF	23.553	22.884
CTRB	64	S	CAMK/CASK	IKKKELNSP QMTPF	4.136	3.657
CTRB	79	S	CAMK/CASK	KKFNE ISLLESN**	4.347	3.657
CTRB	64	S	CAMK/DAPK	IKKKELNSP QMTPF	11.805	9.755
CTRB	64	S	CAMK/PIM	IKKKELNSP QMTPF	16.521	15.765
CTRB	83	S	CAMK/PKD	E ISLLESN******	22.885	21.331
CTRB	69	T	CAMK/TSSK	LNSP QMTPFKKFNE	4.563	3.512
CTRB	64	S	CK1	IKKKELNSP QMTPF	6.585	6.306
CTRB	69	T	CMGC	LNSP QMTPFKKFNE	43.002	40.815
CTRB	64	S	CMGC/CDK	IKKKELNSP QMTPF	12.353	10.587
CTRB	34	S	CMGC/CDK	PICTFV SPAAQKAF	10.379	6.537
CTRB	69	T	CMGC/CDK	LNSP QMTPFKKFNE	14.171	10.587
CTRB	83	S	CMGC/CDKL	E ISLLESN******	6.119	4.358
CTRB	79	S	CMGC/CK2	KKFNE ISLLESN**	3.412	2.665
CTRB	64	S	CMGC/DYRK	IKKKELNSP QMTPF	18.798	17.356
CTRB	26	S	CMGC/GSK	PLP PPVSPICTFV	9.442	8.674
CTRB	64	S	CMGC/GSK	IKKKELNSP QMTPF	9.247	8.674
CTRB	26	S	CMGC/MAPK	PLP PPVSPICTFV	14.868	12.837
CTRB	64	S	CMGC/MAPK	IKKKELNSP QMTPF	12.865	12.837
CTRB	47	S	CMGC/SRPK	AFQP PRSCGTKYET	390.512	319.434
CTRB	64	S	TKL	IKKKELNSP QMTPF	5.474	5.299
CTRB	26	S	TKL/RAF	PLP PPVSPICTFV	2.504	2.477
CTRB	16	S	Othar/CAMKK	RA LDFLSRLPLP P	4.31	3.05
CTRB	16	S	Othar/Haspen	RA LDFLSRLPLP P	2.495	0.998
CTRB	79	S	Othar/IKK	KKFNE ISLLESN**	11.146	8.821
CTRB	64	S	Othar/MOS	IKKKELNSP QMTPF	3.158	2.885
CTRB	79	S	Othar/MOS	KKFNE ISLLESN**	3.299	2.885
CTRB	83	S	Othar/MOS	E ISLLESN******	3.219	2.885
CTRB	64	S	Othar/PEK	IKKKELNSP QMTPF	2.369	1.697
CTRB	69	T	Othar/PEK	LNSP QMTPFKKFNE	3.724	1.697
CTRB	79	S	Othar/PEK	KKFNE ISLLESN**	5.343	1.697
CTRB	83	S	Othar/PEK	E ISLLESN******	5.03	1.697
CTRB	79	S	Othar/PLK	KKFNE ISLLESN**	15.091	14.476
CTRB	16	S	Othar/TLK	RA LDFLSRLPLP P	2.318	2.277

CTRB	69	T	Othar/TLK	LNSP QMTPFKKFNE	2.375	2.277
CTRB	79	S	Othar/TOPK	KKFNE ISLLESN**	3.683	3.19
CTRB	79	S	Dual/TK/Lumr	KKFNE ISLLESN**	6.285	5.599
CTRB	16	S	Dual/TK/Src	RA LDFLSRLPLP P	4.121	4.017
CTRB	64	S	Dual/TK/Src	IKKKELNSP QMTPF	4.202	4.017

Supplementary Table 2: Oligomers and DNA substrates used in this study

Oligo- H3	5' -TTGATAAGAGGTCATTTGAATTCATGGCTTAGAGCTTAATTGCTGAATCT GGTGCTGGGATCCAACATGT TTTAAATATG
Oligo- H4	5'- CATATTTAAAACATGTTGGATCCCAGCACCAGATTCAGCAATTAAGCTCT AAGCCATGAATTCAAATGAC CTCTTATCAA
Oligo- H5	5'- CATATTTAAAACATGTTGGATCCCAGCACCAGATTCAGCATACGTTACCG ATCGTACGTT CGATGCTGGC TACTGCTAGC
Oligo-2040	5'-AAATGAACATAAAGTAAATAAGTATAAGGATAATACAAAATAAGTAAATG AATAAACATAGAAAATAAAGTAAAGGATATAAA
Oligo-2041	5'TTTATATCCTTTACTTTATTTTCTATGTTTATTCATTTACTTATTTTGTATTAT CCTTATACTTATTTACTTTATGTTCATTT
Oligo-A	5'-TCTTATTTATGTCTCTTTTATTTCATTTCCTATATTTATTCCTATTATGTTTTATT CATTTACTTATTCTTTATGTTCATTTTTTATATCCTTTACTTTATTTTCTCTGTTTAT TCATTTACTTATTTTGTATTATCCTTATCTTATTTA
Oligo-B	5'- TAATACAAAATAAGTAAATGAATAAACAGAGAAAATAAAG
Oligo-C	5'- CTTTATTTTCTCTGTTTATTCATTTACTTATTTTGTATTA
Oligo-12mer	5'- GGCTATGCG TTA
80bp dsDNA	Annealed product of 5'-^{32}P- (or Cy5-) labeled Oligo-H3 and Oligo-H4
83bp dsDNA Trap	Annealed product of Oligo-2040 and Oligo-2041
40mer dsDNA	Annealed product of 5'-^{32}P- labeled Oligo-B and Oligo-C
Fork substrate	Annealed product of 5'-Cy5- labeled Oligo-H3 and Oligo-H5

Supplementary References

1. Marsh, J.A., Singh, V.K., Jia, Z. & Forman-Kay, J.D. Sensitivity of secondary structure propensities to sequence differences between alpha- and gamma-synuclein: implications for fibrillation. *Protein Sci* **15**, 2795-804 (2006).
2. Esashi, F. et al. CDK-dependent phosphorylation of BRCA2 as a regulatory mechanism for recombinational repair. *Nature* **434**, 598-604 (2005).
3. Wang, C. et al. GPS 5.0: An Update on the Prediction of Kinase-specific Phosphorylation Sites in Proteins. *Genomics Proteomics Bioinformatics* **18**, 72-80 (2020).
4. Zhao, Q. et al. GPS-SUMO: a tool for the prediction of sumoylation sites and SUMO-interaction motifs. *Nucleic Acids Res* **42**, W325-30 (2014).
