# Supplementary material for: DNA binding and RAD51 engagement by the BRCA2 C-terminus orchestrate DNA repair and replication fork preservation
Source: Nat Commun. 2023 Jan 26;14:432. doi: 10.1038/s41467-023-36211-x (PMC9879961; doi:10.1038/s41467-023-36211-x)
Supplement: Supplementary file 3 — Reporting Summary [file 41467_2023_36211_MOESM3_ESM.pdf]

nature portfolio

Corresponding author(s): Patrick Sung

Last updated by author(s): 2023-1-3

Reporting Summary

Nature Portfolio wishes to improve the reproducibility of the work that we publish. This form provides structure for consistency and transparency in reporting. For further information on Nature Portfolio policies, see our Editorial Policies and the Editorial Policy Checklist.

Statistics

For all statistical analyses, confirm that the following items are present in the figure legend, table legend, main text, or Methods section.

n/a	Confirmed	
☐	☒	The exact sample size (*n*) for each experimental group/condition, given as a discrete number and unit of measurement
☐	☒	A statement on whether measurements were taken from distinct samples or whether the same sample was measured repeatedly
☐	☒	The statistical test(s) used AND whether they are one- or two-sided<br>*Only common tests should be described solely by name; describe more complex techniques in the Methods section.*
☒	☐	A description of all covariates tested
☐	☒	A description of any assumptions or corrections, such as tests of normality and adjustment for multiple comparisons
☐	☒	A full description of the statistical parameters including central tendency (e.g. means) or other basic estimates (e.g. regression coefficient) AND variation (e.g. standard deviation) or associated estimates of uncertainty (e.g. confidence intervals)
☐	☒	For null hypothesis testing, the test statistic (e.g. *F*, *t*, *r*) with confidence intervals, effect sizes, degrees of freedom and *P* value noted<br>*Give P values as exact values whenever suitable.*
☒	☐	For Bayesian analysis, information on the choice of priors and Markov chain Monte Carlo settings
☒	☐	For hierarchical and complex designs, identification of the appropriate level for tests and full reporting of outcomes
☒	☐	Estimates of effect sizes (e.g. Cohen's *d*, Pearson's *r*), indicating how they were calculated

Our web collection on statistics for biologists contains articles on many of the points above.

Software and code

Policy information about availability of computer code

Data collection	Phosphorimaging was done with Biorad Personal Imager FX. Western blot images were acquired using Biorad ChemiDoc MP. SDS -PAGE gelimages stained with Coomassie blue were acquired with Biorad ChemiDoc MP or Epson Perfection V700 Photo Scanner. RAD51 foci and DNA fibers were imaged on a Zeiss Axio-Imager Z2 microscope equipped with ZEN Blue software (Carl Zeiss Microscopy). % GFP cells were measured using LSRII (BD Biosciences). CD spectrum was obtained with JASCO J810 spectropolarimeter. NMR spectra were obtained with an Agilent DD2 800 MHz spectrometer
Data analysis	Phosphorimaging analysis was performed with ImageQuant v8.2 or Image lab 5.2.l. Western blot and Coomassie blue gels images were processed with Image lab 5.2 or Epson ScanSmart. NMR data were processed with Vnmrj in Agilent BioPack. RAD5l foci and DNA fibers where assessed using Fiji and ImageJ v1.53. % GFP cells were analyzed with FlowJo v10 software (BD Biosciences). Data analysis and graphs were plotted using Graph Pad Prism 8.4 or Microsoft Excel 16

For manuscripts utilizing custom algorithms or software that are central to the research but not yet described in published literature, software must be made available to editors and reviewers. We strongly encourage code deposition in a community repository (e.g. GitHub). See the Nature Portfolio guidelines for submitting code & software for further information.

Data

Policy information about availability of data

All manuscripts must include a data availability statement. This statement should provide the following information, where applicable:

- Accession codes, unique identifiers, or web links for publicly available datasets
- A description of any restrictions on data availability
- For clinical datasets or third party data, please ensure that the statement adheres to our policy

All data generated or analyzed during this study are included in the article and its supplementary information. The NMR data generated in this study have been deposited in the Biological Magnetic Resonance Bank (BMRB) under accession number 51679. Source Data are provided with this paper.

Human research participants

Policy information about studies involving human research participants and Sex and Gender in Research.

Reporting on sex and gender	This study did not involve any human participants.
Population characteristics	This study did not involve any human participants.
Recruitment	This study did not involve any human participants.
Ethics oversight	This study did not involve any human participants.

Note that full information on the approval of the study protocol must also be provided in the manuscript.

Field-specific reporting

Please select the one below that is the best fit for your research. If you are not sure, read the appropriate sections before making your selection.

☒ Life sciences ☐ Behavioural & social sciences ☐ Ecological, evolutionary & environmental sciences

For a reference copy of the document with all sections, see nature.com/documents/nr-reporting-summary-flat.pdf

Life sciences study design

All studies must disclose on these points even when the disclosure is negative.

Sample size	Sample sizes were chosen based on our previous experience to obtain statistical significance and reproducibility. Exact sample sizes are included in the paper.
Data exclusions	Data were excluded from analysis when control samples didn't work. Occasionally samples which are not associated with the study were examined at the same time and the data obtained from the samples were excluded for in the paper.
Replication	All findings were repeated as indicated in the paper and all the replication attempts were successful.
Randomization	Randomization was not part of the study design. In vitro assay samples were assigned to experimental in a non-biased way
Blinding	Blinding method was performed for image acquisition and analysis during microscopy. Blinding was not practically feasible to carry out during the other experiments in this study, and it would not be expected to impact the results.

Reporting for specific materials, systems and methods

We require information from authors about some types of materials, experimental systems and methods used in many studies. Here, indicate whether each material, system or method listed is relevant to your study. If you are not sure if a list item applies to your research, read the appropriate section before selecting a response.

Materials & experimental systems

n/a	Involved in the study
☐	☒ Antibodies
☐	☒ Eukaryotic cell lines
☒	☐ Palaeontology and archaeology
☒	☐ Animals and other organisms
☒	☐ Clinical data
☒	☐ Dual use research of concern

Methods

n/a	Involved in the study
☒	☐ ChIP-seq
☒	☐ Flow cytometry
☒	☐ MRI-based neuroimaging

Antibodies

Antibodies used

Primary Ab:
α-MBP antibody (Novus Biologicals, Cat. # NB100-66609H), Western blot (WB), (1: 1000)
α-Tubulin antibody (Cell signaling Technology, Cat. # 2125), WB, (1: 3000)
α-Histone H3 antibody (Cell signaling Technology, Cat. #5192), WB, (1: 1000)
α-BRCA2 antibody (Millipore Sigma, Cat. # OP95), WB, (1:500)
α-RAD51 antibody (Abnova, Cat. #: H00005888-B01P), WB, (1:500)
α-RAD51 (H-92; Santa Cruz Biotechnology, Cat. # sc-8349), Foci analysis, (1:1000)
α-BrdU (Biorad, Cat #MCA2060T), DNA fiber analysis, (1:100)
α-BrdU (Beckton Dickinson, Cat #347580), DNA fiber analysis, (1:100)

Secondary Ab:
α-mouse-IgG-HRP (Pierce Cat. #31450), WB, (1:4000)
α-rabbit-IgG-HRP (Sigma #A6154), WB, (1:4000)
Alexa Fluor 488 goat α-mouse IgG (Thermo Fisher Scientific, Cat #A-11029), DNA fiber analysis (1:300)
Alexa Fluor 594 goat α-rat IgG (Thermo Fisher Scientific, Cat #A-11007), DNA fiber analysis, (1:300)
Alexa Fluor 594 goat α-rabbit IgG (Thermo Fisher Scientific, Cat #A-11037), RAD51 foci analysis (1:750)

Validation

α-BRCA2 antibody (Millipore Sigma, Cat. # OP95) was validated by WB of cell extracts in HeLa U2OS from our lab (Ref 10 in the manuscript: Zhao et al. Mol Cell 2005, and in this paper (Sup. Figure 4), MCF-7 (PMID: 9619832), and 239T cells (PMID:16793542, 22293751).
α-Tubulin antibody (Cell signaling Technology, Cat. # 2125) was validated by WB analysis of extracts from C6, COS-7, NIH/3T3 and HeLa cells, by immunohistochemical analysis of human glioblastoma, confocal immunofluorescent analysis and by flow cytometric analysis of HeLa cells (validation information on the manufacturer's website)
α-MBP antibody (Novus Biologicals, Cat. # NB100-66609H) was validated by western blot (WB) in our lab and the manufacturer (Novus Biologicals) by testing specific recognition of expression of MBP-tagged proteins from extracts of Hi5 insect cells, E coli, and DLD, HeLa, U2OS, and testing MBP-tagged purified proteins.
α-Histone H3 antibody (Cell signaling Technology, Cat. #5192) was validated by WB of extracts from C2C12, 293, COS, PC12 and HeLa cells (validation information on the manufacturer's website)
Rabbit α-RAD51 antibody (Abnova, Cat. #: H00005888-B01P) was validated by WB of extracts in human colon, PC-12. Raw 264.7. Jurkat. NIH/3T3, 293T, and by immunohistochemistry of human tissue (validation information on the manufacturer's website)
Rabbit α-RAD51 for foci detection (H-92; Santa Cruz Biotechnology, Cat. # sc-8349), (1:1000) was validated by RAD51 foci analysis in HeLa from our lab (Ref 9: Zhao, W. et al. Nature 550, 360-365 (2017) and this paper (Sup. Figure 4).
Rat α-BrdU (Bio-Rad, Cat #MCA2060T) was validated by immunocytochemisty staining in studies published in PMID: 27768761, 25628356, 21295139, 27213272. for CldU detection (PMID: 34107305, 29038466)
Mouse α-BrdU (Beckton Dickinson, Cat #347580) was validated by intracellular staining and immunohistochemistry fiber analysis in study published in PMID: 6577444, PMID: 3582069, and by DNA fiber analysis for IdU detection (PMID: 34107305, 29038466)

Eukaryotic cell lines

Policy information about cell lines and Sex and Gender in Research

Cell line source(s)

DLD1 cells (BRCA2-/-): Horizon Discovery HD 105-007: provided by Dr. Ryan Jensen at Yale University.
HeLa Dr-GFP cells: Dr. Jeffrey Parvin at Ohio State University (PMID: 20103620)
Sf9 cells (Thermo Fisher Scientific, Cat #B825-01)
Hi-Five cells (Thermo Fisher Scientific, Cat #B855-02)

Authentication

DLD1 cells (BRCA2-/-) were identified by loss of BRCA2 by western blot analysis. HeLa-DR cells were identified by GFP signal upon I-SceI expression.
Sf9 and Hi-Five cells were validated based on their features (generating baculovirus and over-expressing proteins).

Mycoplasma contamination

We performed routine mycoplasma tests using MycoAlert Mycoplasma detection Kit (#LT07-318) and confirmed that all cell lines tested negative for mycoplasma contamination.

Commonly misidentified lines
(See ICLAC register)

Not used in this study.
